# The Hospital. Nursing Section

**Published:** 1904-12-24

**Authors:** 


### The Hospital.

### Nursing Section.

Contributions for this Section of "The Hospital" should be addressed to the Editor, "The Hospital," Nursing Section, 28 & 29 Southampton Street, Strand, London, W.C.

No. 952.—Vol. XXXVII. *SATURDAY, DECEMBER* 24, 1904.

### Notes on News from the Nursing World.

### OUR CHRISTMAS CLOTHING DISTRIBUTION.

The whole of the contributions to our Christmas clothing distribution were on view at the offices of The Hospital last Tuesday afternoon, and immediately after the exhibition was over parcels were despatched to the various hospitals. Although there were several handsome and acceptable gifts, most of them hand made, the parcels were not quite so numerous as last year, possibly owing to the fine autumn. There was a good parcel from Nurse Cecil who had been helped in the making of some clothes by an old patient. Miss Henslowe sent men's socks, children's petticoats and babies' wool boots, all knitted or crocheted. One of the largest parcels from Miss Taylor, included two useful shawls. Two warm crochet shawls, contributed by Nurse Sneath, "with the sender's love and best wishes to two old ladies," will be real comforts to some hospital "grannies." Other useful contributions were dressing-jackets, various knitted garments, baby clothes, warm underclothing, a woman's warmly lined dress, and a number of woollen vests. Of all women, nurses must realise how much such gifts are needed and appreciated in charitable institutions. Hospital nurses have scanty time for needlework, and what they have they naturally like to devote to sewing for their own institutions; but, surely, some private nurses might spare a little more time for making clothes for this charity? Convalescent patients, too, can often be induced to take a practical interest in the clothing distribution, and if lay readers only realised the poverty of some of the patients in the London Hospitals at the present moment, they, too, would help, however little. Therefore, in order that our clothing distribution may be more satisfactory next year, we beg our readers to begin sewing and knitting again as soon as possible, while they are still conscious of the need. We have to acknowledge the receipt of parcels from Nurse Sneath, Miss L. Altree, Sydenham; K. C. G., Penrith; Miss Pace, Lower Faldonside, near Melrose; Policy Holder 3521; and Mrs. Binnie. The Editor of The Hospital had pleasure in contributing a useful bundle of warm clothing for men's wear.

### THE RESPONSE TO A CRY FROM INDIA.

It will be recollected that a fortnight ago we mentioned that Mrs. Creighton had stated that she had appealed in vain to English nurses to go out to India and undertake the duty of helping to train native women as nurses. We expressed regret at her want of success, and our conviction that the mention of the need in our columns would probably bring volunteers for the work. Our expectation has been fulfilled, and in addition to a very large number of letters which have reached this office, Mrs. Creighton herself has received several communications. In the report of the meeting at which her address was delivered, it was not intimated that her appeal for nurses was made in the interests of the Society for the Propagation of the Gospel, and not on her own account. The Society are still, we are officially informed, in want of missionary nurses, and we have forwarded to Miss Mackenzie, the Secretary of the Women's Work Committee of the Society for the Propagation of the Gospel, 19 Delahay Street, Westminster, S.W., all the applications which have come into our possession, and all inquiries in respect to the subject. There are openings for the right kind of women in India, and possibly in South Africa and Borneo; but it is useless for any one who is not qualified and prepared to act as a nurse-missionary to write to Miss Mackenzie.

### A SANATORIUM UNDER CARETAKERS.

A surprising experience recently occurred to a temporary night nurse in a Lancashire sanatorium. The institution had a nurse-matron in charge and a night nurse, engaged by the medical officer of health. There were also attached to it a caretaker and his wife, who lived in their own apartments in the administrative block, but were allowed by the Urban District Council Committee to use the nurses' bathroom. This was resented by the nurses, and, on the other hand, the caretakers strongly objected to any interference with their work on the part of the nurse-matron, while the wife did not like the stores being locked up under the matron's care. Naturally, there was friction, and the committee preferred that the nurses should resign rather than that the caretakers should leave in less than three months. They should have insisted upon the caretakers being absolutely amenable to the authority of the nurse-matron.

### THE END OF THE CROYDON CONTROVERSY.

At the meeting of the Croydon Guardians on Tuesday, Dr. W. B. Addison moved the resolution restoring the matron's certificate to the training certificates of probationers in the infirmary. The motion was seconded by Miss Musselwhite, and a discussion of some length followed, in the course of which it was urged that Dr. Addison's statement that the nurses had acted entirely on their own initiative was open to question, and an extract from The Hospital was read in order to show that outside influences had been brought to bear. Mr. Owen said he did not think that anyone would doubt but that the board could trust the present matron, Miss Pagen, but he pointed out that she might seek a position elsewhere and be followed by someone whom they could not trust. At any rate, to save the board's position, he proposed to add to the resolution of Dr. Addison, the words, "Provided, at any future time, circumstances shall arise which in the opinion of the Board shall make it necessary to dispense with the matron's signature on the certificates then the Board shall be at liberty forthwith to dispense with the same." In reply to a question the Clerk said the effect of this addition would be that at any future time if the Board wished to dispense with the matron's signature they could do so without applying to the Local Government Board and without rescinding any previous resolutions. The addendum having been seconded, and Dr. Addison and Miss Musselwhite having accepted it as part of the original resolution, the latter as altered was put to the vote. Eighteen voted for it and six against. It was therefore declared carried, and in spite of the addition which was made to save the Board's position, we do not think that the Croydon Guardians will ever again attempt to dispense with the matron's signature to the certificate of their training school. If the matron cannot be trusted to sign the certificate she ought not to be appointed matron.

### REFORMS NEEDED AT CHELTENHAM HOSPITAL.

There are two matters that call for criticism in the account of the conditions of nursing at Cheltenham General Hospital, which is given by our commissioner in another part of the paper to-day. The nurses, both in the hospital and in the home for the private nurses, sleep in cubicles. This, although the cubicles are comfortably furnished, is a serious drawback which cannot be remedied too soon. Another disadvantage is that the meals of the nurses are served in their ordinary sitting-room. It appears that lack of space is the reason for these arrangements, but in a prosperous town like Cheltenham it should not be difficult to raise sufficient money to enable the hospital authorities to provide proper accommodation for the staff. We observe that there is "an up-to date little operating-theatre"; the provision for the health of the nurses ought also to be up to date.

### CONSUMPTIVES ON BOARD SHIPS.

In reply to the circular issued by Sir William Broadbent, the Chairman of the National Association for the Prevention of Consumption, inviting the steamship authorities to observe precautions against the spread of infection from persons suffering from tuberculosis on board, it is affirmed by some of the companies that adequate regulations are already in force. But this is an excellent reason why a trained nurse should be regarded as necessary to the proper equipment of an ocean-going steamer. She might in many cases be able to detect the presence of consumptives even before the malady had reached the advanced stages, to quietly provide means of disinfection, enforce the regulations, and in various ways to minister to the comfort of the sufferers. We do not, in fact, see how the danger which Sir William Broadbent has pointed out can be sufficiently guarded against unless the services of a trained nurse are called into requisition.

### SAVING THE COST OF A TRAINED NURSE.

At the last meeting of the Braintree Guardians the master of the workhouse reported that the wife of the labour master had volunteered to nurse the fever cases in the house during the recent outbreak. In recommending that she should be remunerated for her services, and compensated for articles of clothing which she had sacrificed in her work, he said that she had saved the board about £24, "the cost of a trained nurse." The Guardians decided to make her a grant of £5, and to replace the clothing destroyed. The remuneration does not err on the side of generosity. But, of course, the services of the wife of the labour master, who is employed as children's caretaker, should not have been accepted in lieu of those of a trained nurse, either on the ground of economy or for any other reason. If, in the circumstances, there had been any deaths from fever, the Guardians might have been in an awkward position.

### THE HOLIDAYS OF DISTRICT NURSES.

The Superintendent of Queen's Nurses at Leeds calls attention in our columns to-day to the difficulties attending the provision of suitable holidays for the staff of the District Association. We gladly insert her letter, which will, doubtless, have the desired effect of eliciting the opinions of persons possessed of experience in district nursing. But we certainly cannot support the proposal of a subscriber to the Leeds Association that the whole of the nurses should take a holiday of four weeks simultaneously in the summer. It may be quite true that the work at that period is the lightest, but, in the best of weather, there are always sick persons, and the idea of leaving them to shift for themselves should at once be dismissed. The argument that the absence of the nurses *en bloc* would facilitate cleaning operations at the home is not convincing, but why should not the nurses go in two or more detachments?

### A CONTINENTAL ERROR.

It appears that abroad there are people under the delusion that trained nurses in this country are registered. One of the hospitals on the Continent advertises for the services of an English nurse, who, in addition to being "well bred and mannered," must be "registered." No doubt the certificate of a recognised training-school will be considered as satisfactory as a certificate of registration.

### ROYAL COMPLIMENT TO A CRIMEAN NURSE.

On the occasion of the visit of the King and Queen to St. Mary Church, Bury St. Edmunds, on Saturday some of the old inhabitants of the town were allowed to sit in the church. Among these was Nurse Langley, who went out with Miss Nightingale to the Crimea, and their Majesties, being informed of her identity, afforded the venerable lady much pleasure by entering into conversation with her.

### PRINCESS CHRISTIAN AND THE MOTHERS' AID SOCIETY.

On Saturday Princess Christian visited Queen Charlotte's Hospital and presided at the meeting of the Mothers' Aid Society, which assists the more deserving amongst the patients, both in and out, after their confinement. She was received by Mr. H. A. Harben, vice-chairman. Her Royal Highness expressed her deep sympathy with the work of the hospital and of the Mothers' Aid Society, and said she hoped to come again and make a thorough inspection of the wards.

### SCOTTISH QUEEN'S NURSES AND EDINBURGH INFIRMARY.

At the annual meeting of the subscribers and friends of the Scottish branch of Queen Victoria's Jubilee Institute for Nurses the Lord Provost expressed his deep regret that the last nurse had ceased to be trained at the Edinburgh Royal Infirmary. Until now twelve of the Queen's nurses had been sent from the Scottish Institute to the Infirmary for hospital training, and he intended as chairman of the Infirmary Board to thoroughly inquire into the matter and ascertain why a privilege which was much valued had been withdrawn. He thought that the arrangement was for the benefit of the Infirmary, as well as for that of the Institute, and for that of the people generally. The chairman then paid a high tribute to the Queen's nurses in Scotland, a record of whose work is given in detail in the report. The excess of expenditure over receipts was nearly £100, and it was explained that the Council of the Institute were only precluded from meeting an urgent demand for additional nurses in Edinburgh owing to inadequacy of funds.

### QUEEN ALEXANDRA'S MILITARY NURSING SERVICE.

We are officially informed that 16 new staff nurses have been appointed to Queen Alexandra's Imperial Military Nursing Service. Miss K. A. Allsop, Miss K. Coxon, Miss E. St. Quintin, and Miss L. Strickland have been posted to Aldershot; Miss M. Clements, Miss H Hare, Miss D. Michell, Miss J. Murphy, and Miss B. Rankin, to Colchester; Miss A. Rowe, to Portsmouth; Miss P. Steele, to the Royal Military College, Sandhurst; Miss E. M. Walby, and Miss V. C. Paschall, to Chatham; Miss L. Cunningham, to Woolwich; Miss S. N. Daly, to Hounslow; and Miss H. L. A. Jack, to Alton. The appointments of Miss L. M. Moor, Miss M. E. Richardson, and Miss A M. Pagan, as staff nurses, have been confirmed. Staff Nurses M. L Harris, K. M. Hewetson, L. E. Mackay, E. S. Mason, and M. Walker, have been promoted to be sisters. Several changes of station have been made among matrons. Miss L. Hardement has been sent to Chatham; Miss M. C. S. Knox, R.R.C., has been transferred from Dover to South Africa; Miss C. H. Potts, from Curragh to South Africa; and Miss H. W. Reid, from Wynberg to Pretoria. Sister E. Beck has been transferred from York to South Africa; Sister E. H. Hay, from Alton to Colchester; Sister L. M. Lyall, from Woolwich to South Africa; Sister E S. Mason, from Aldershot to Colchester; Sister R. Osborne, from Alton to South Africa; Sister K. Pearse, from Aldershot to Chatham; Sister C. K. E. Steel, from Aldershot to London; and Sister E. O. Stewart, from Aldershot to South Africa.

### SIR WILLIAM THOMSON AND THE IRISH NURSES' ASSOCIATION.

A lecture was delivered on Tuesday last week at the house of the Irish Nurses' Association in Lower Leeson Street, Dublin, on "The Theory and Practice of Cleanliness in Surgery," by Sir Wm Thomson, C.B., F.R.C.S. Miss MacDonnell, R.R.C., lady superintendent of the Richmond Hospital, was in the chair. The subject is one of absorbing interest to all nurses, and Sir William's graphic description of surgery under the old *régime*, as compared with the surgery of to-day and the practical lessons to be derived therefrom, was listened to with the keenest attention by the members, of whom there was a large attendance. At the close of the lecture a hearty vote of thanks to Sir Wm. Thomson was carried by acclamation.

### THE NORLAND INSTITUTE.

The quarterly "At Home" of the Norland Institute was held at Pembridge Square last Saturday afternoon. A large number of people were present, including some past nurses and their little charges, present nurses and their friends. The foundress, Mrs. Ward, gave an address, mainly to the probationers who had just finished their course at the Institute; and, judging by the happy looks of the nurses and the interested expressions of the visitors, an enjoyable afternoon was spent. The Institute was open for inspection, and many persons were shown over it. Specimens of the probationers' Kindergarten work were on view, including some excellent clay modelling. The needlework, too, was good; and, for amateur laundresses, the probationers' laundry work seemed very fair indeed. An exhibition of interest was a collection of Kindergarten work: the result of the labours, under the superintendence of Norland nurses, of children in their own home nurseries

### SHAMPOO-ROOMS FOR NURSES.

An excellent hygienic arrangement at the Norland Institute is a shampoo-room—or, to be exact, cubicle—for the nurses. It is fitted with the orthodox basin and hose. This idea, which has been adopted at some of the nurses' clubs, might successfully be copied by many hospital authorities in their nurses' homes. Having regard to the scanty leisure of nurses, it is of great importance that they should have a facility of this kind, and the other points in favour of such a convenience are obvious.

### THE BLOOMSBURY EXPLOSION.

It was stated in the daily Press that the gas explosion in Bloomsbury greatly frightened the children in the Great Ormond Street Hospital. As a matter of fact, however, all that happened was that the shock of the explosion was felt in the hospital. The pacification of the little patients, which was attributed to the nurses, was not therefore required. We have no doubt that if it had been, the nursing staff would have been equal to the demands upon them.

### EXAMINATION OF BETHNAL GREEN INFIRMARY NURSES.

Miss Dodds, matron of the Bethnal Green Infirmary, has just completed a series of lectures on Nursing to thirty probationers, and a written examination was held on Tuesday last week. Twenty-eight passed successfully. The honours were taken by Nurses Sandy, Angel and McDonald, Bothwick and Whitman, and Eastty.

### SHORT ITEMS.

In consequence of the Christmas holidays the offices of the Royal National Pension Fund for Nurses, 28 Finsbury Pavement, will be closed on Monday and Tuesday next.—A most enjoyable entertainment was given to the in-patients of the Cancer Hospital, London, on Thursday evening, last week, by Miss Bessle's Comedy Company.

### The Nursing Outlook.

"From magnanimity, all fear above;
From nobler recompense, above applause,
Which owes to man's short outlook all its charm."

### LEVELLING UP THE MIDWIFE.

The time of grace during which the uncertified midwife can claim registration is rapidly drawing to a close, and in three months more it will be possible to form an estimate of the number of *bona fide* midwives of the old-fashioned type still following their calling in this country. It will not be denied that these persons, as a body, have, on the one hand, acquired considerable dexterity in the performance of their duties, and, on the other hand, that they are lamentably ignorant of certain precautions which, within the last few years only in the history of the world, have been found to be necessary for the safety alike of mother and child. It is in the highest degree desirable that all uncertified women who propose to practise under the Act should be brought under instruction, and a useful step in this direction has been taken by the issue of a syllabus of lectures to *bona fide* midwives on the Midwives Act, and the rules of the Central Midwives Board, by the Association for promoting the Training and Supply of Midwives. Following these rules point by point with suggestions for amplification on the part of the teacher, the syllabus is an excellent synopsis of antiseptic midwifery to which nothing need be added, and from which nothing could with safety be omitted. We deprecate, however, the expression "lectures" in this connection. The class of women for whom they are intended are not likely to derive much benefit from a stream of talk, however dexterously administered. But if gathered round a table in a class-room, they could be taught by means of questions and answers by a friendly homely teacher, many unsuspected misapprehensions would be brought to the surface and melt away. In such classes, the first aim of the teacher should be to ascertain how far her pupils are already on the right track, and in what particulars their procedure is wrong, and to this end a private talk with each member of the class is essential.

The main difficulty is, of course, to get the women together for instruction. In towns it may be quite possible to arrange small classes by personally inviting those who are practising midwifery to attend classes arranged for their benefit. But no initiative on the part of the midwives themselves must be expected. A measure of hostility to the "new-fangled" ways is natural and will take a long time to overcome.

In rural districts the difficulties in the way of instructing midwives are much more serious. In the first place a large proportion of the rural midwives are abstaining altogether from claiming registration. The formidable-looking circular in long envelope which was sent round to all the midwives of the country early in the present year has had the effect in countless instances of checking their practice once and for all. After showing it round to all her relatives, to the parson, and to the district lady, the rural midwife has generally reached the conclusion that there are too many pitfalls in midwifery to make it a safe calling for her to follow. Her gains are probably not more than £5 a year, or possibly in the neighbourhood of a large village, as much as £10. She will either do without these emoluments or adopt some safer industry.

It may be argued that this was to be desired. There is, however, some danger of overlooking the necessities of the rural poor. It can never pay a woman who has undergone expensive training, to settle down as midwife in a scantily-populated district; the extent of its district must be limited by her powers of endurance, and even cycling has not made it possible for one woman to serve a neighbourhood covering a radius of more than four or five miles from her residence. This cannot give her a living. Must midwifery in rural districts in the future of necessity partake of an eleemosynary character? At the lowest estimate a district nurse must receive from £50 to £60 a year. At present they are a handful among the rural population in England. Has any one counted the cost of covering the country with a network of subsidised midwives to take the place of the ignorant but generally skilful "wise women," who are now discontinuing their duties among the labourers' wives?

We are not now concerned with the additional ill-remunerated labour which must fall on the parish doctors. Delivery by a doctor leaves the mother still under the necessity of procuring attendance before and after labour, when dirt and ignorance may still do their baleful part.

We cannot but think that some effort should be made before it is too late to retain for the service of the poor, many of the rural midwives who have been in reputable practice and to give them instruction on some such lines as are indicated in the above syllabus. It is hard to say on whom this duty should devolve. We believe that the district councils would find it the truest economy in the end to appoint a trained nurse inspector who would personally visit and advise with such midwives as have hitherto failed to apply for registration and who would ultimately arrange for their instruction by twos and threes in the rules of the Central Midwives Board.

### Lectures Upon the Nursing of Mental Diseases.

By Robert Jones, M.D.Lond., B.S., F.R.C.S.Eng., M.R.C.P.Lond., Resident Physician and Superintendent of the London County Asylum, Claybury.

LECTURE XXII. (*continued from page* 135.)

Whether or not it be due to the fact that the skin is developed from precisely the same area and structure which gives rise to the organ of mind—the brain—I consider that this tissue of the body, *i.e.*, the skin in the insane, undergoes marked changes, and is more liable to certain abnormal conditions and unhealthy activities than are ordinarily met with in sane people. All medical men and nurses who have much to do with insane persons know that there is a peculiar odour attaching to these persons when in the acute stage, and, in consequence, to all institutions for mental cases unless very efficiently ventilated. The whole of the activities of the skin are under the reflex influence of the nervous system, and when the latter is affected, altered function results in whatever organ comes under its control. In regard to the proper supervision of the skin, and also of the hair of insane patients, it can truly be stated that no other duty is more trying to the nurse. To keep the restless patients tidy, the demented ones clean, and the melancholy from obstinate self-neglect, are very constant duties, and as trying as they are constant. The hair, especially among females of the poorer classes, is often loathsomely dirty when they are received into asylums, and the rules of the establishment do not permit of the hair being cut. In consequence the nurse has most repellent work to undertake, and for the quiet, unostentatious performance of her duties without grumbling, she deserves and needs every kindly encouragement that can be bestowed upon her by those in authority over her. I have elsewhere stated that were the devotions of the nurse not charitable and self-denying in the extreme, and were they not rewarded by the high ideal sense and the honour of serving others, there could be no adequate compensation for her repugnant duties in an asylum for the poor. That these are often thankless, owing to the mental infirmity of the sufferer, is why this kind of service is unpopular among ladies of private means who in other places find gratitude a sufficient reward for self abnegation. No monetary consideration can be an equivalent recompense for the nurse's devotion and attentions to such cases. In the *toilette*, and as an application for effectively removing the ova or "nits" attached to the hair, the medical officers generally order a special lotion of eucalyptus and ether in equal parts which the nurse pours into a dish, soaks up with a pledget of cotton wool or lint and applies to a strand of the hair, thoroughly rubbing it up and down until all the hair has been carefully gone through and cleansed. After the application of the eucalyptus is completed some white precipitate ointment is used with a few drops of lavender essence or bergamot to disguise its significance. This is for the purpose of destroying the living parasites, and the whole of this treatment is repeated day by day until the hair at the end of a week or perhaps several weeks is clean. It is painful to know that not only is the hair of the head thus neglected but also the hair on the body and not infrequently the eyelids and eyelashes as well. The neglect which permits women to get into such a repugnant state not infrequently originates in drink, followed as it invariably is by squalid poverty and disease, conditions which are important factors in physical deterioration. Words fail adequately to describe the pitiful condition of these persons, words equally fail to express the admiration which self-denying women command for their noble and high altruism, as nurses upon them.

Another form of parasite, but of a vegetable kind, which affects the hairy surface and which has to be dealt with is ringworm—*tinea tonsurans*. It attacks the scalp of men and women, the beards of men (*sycosis*), and the bodies of both. It is extremely difficult to cure and slow to be affected by any remedy. Cleanliness is the first and strictest necessity. Affecting the skin only, is another vegetable parasite (*microsporon furfur*) which appears either as brown patches oftenest on the chest and shoulders, or the front of the abdomen, or it may indeed cover the whole trunk. It is termed *chloasma*, and can easily be cured by rubbing with strong mercurial ointment. A common animal parasite, also indicative of neglect, is the itch (*acarus scabies*), which is equally responsive to treatment; a good hot bath daily with soap and water, and then sulphur ointment well rubbed in afterwards, for a week completely cures this horrible and contagious malady. It is due to a minute creature burrowing under the skin, causing great irritation and not infrequently painful abrasions and pustules. It usually commences in the web between the fingers. *Nettlerash* occurs among the insane, and is either caused by dietary indiscretions or through idiosyncrasy in relation to special articles of food, such as fish, shell-fish, or strawberries.

The other so-called rashes, which I think are more common among the insane are the different kinds of *herpes*, which consists of an eruption of small vesicles arranged in groups on an inflamed base. These vesicles run their course and are not succeeded by fresh groups, but there is often a feeling of irritation, tension, burning, or neuralgic pains preceding and following their appearance. When herpes girdles the trunk unilaterally it is called *herpes zoster* or shingles. It follows the course of a definite nerve, and it occasionally occurs in cases of general paralysis of the insane, or in excitable and nervous persons. Sometimes it affects the arm, thigh, or face, and it may appear on the lips with an ordinary cold, or in cases of pneumonia.

*Eczema* in its various forms is not infrequently met with in aslyums. It is an inflammatory infiltration of the skin with a tendency to excoriated redness and tending also to the formation of crusts. *Psoriasis* is a scaly skin disease appearing in patches, met with most often on the elbows or knees. *Acne* is a common disfigurement on the face, shoulders, and back. *Lupus* is not uncommonly seen among an asylum population. The various *warts* and *corns*, rashes due to *syphilis*, as also those due to *drugs*, especially the "bromide" rash, are met with; the latter seldom fails to be seen among the epileptic insane. *Maculæ*, *petechiæ*, or *stains*, not removable by pressure on the skin, are sometimes seen as hæmorrhagic spots in cases where the arteries are diseased and tortuous as in senile insanity; also brownish discoloration at the root of the hair and bend of the knees, elbows, etc., in cases of Addison's disease. The *erythema* which indicates rheumatism, chilblains, or *erysipelas* is also seen among the insane. The disfigurement of the ears, through hæmorrhage between the cartilage and the skin is seen among the more demented and chronic class of the insane. Formerly it was an opprobrium to the nurse, but I am quite certain that these *insane ears* are most frequently due to the constant restlessness which marks so many of the chronic insane, who not only strike themselves but also bite, kick, and scratch themselves frequently without obvious intent and for no apparent purpose.

### The Nurses of Cheltenham General Hospital.

INTERVIEW WITH THE MATRON. BY OUR COMMISSIONER.

As I approached the large white square building which gives shelter to those who need surgical and medical attention in Cheltenham I could not help contrasting the position with that of some of the hospitals in our large smoky, crowded towns. All the front windows look over the large football and cricket field belonging to the Boys' College, whilst at the back there is also plenty of fresh air and open space. In the entrance hall stands almost a life-sized statue of the Good Samaritan pouring oil and wine into the wounds of the injured traveller, with the words below "Go and do thou likewise." This was presented to the hospital by one of the senior surgeons who was also vice-president from 1825 to 1854. Miss Möller, the matron, received me in her little office which communicates with her private sitting-room.

I asked her how long she had been at Cheltenham.

"Since June 1900. Before that all my nursing life had been spent at St. Mary's Hospital, Paddington. I was trained there, and for seven years was sister-in-charge of a large male surgical ward."

"I believe you did not find the system of three years' training in force here when you came?"

"No, but it was adopted as soon as possible afterwards—the following October. Probationers are received here a little earlier than at some of the larger hospitals, because, owing to the wards being scattered, we are obliged to keep an ample staff. Therefore the work is not hard. Twenty-two years of age is the minimum."

"Do you find that owing to their youth the nurses suffer at first from ill-health?"

"Not at all. Beside the fact of our not being understaffed, Cheltenham is very healthy; nearly all the nurses keep their bicycles—we have a bicycle-house—and in their off time they can get a good spin along the country roads, which does much to invigorate them."

### Age and Off-Duty Time.

"I think," continued the matron, "you will agree that the off-duty time is on a liberal scale. The nurses are called at 5.45; they breakfast at 6.30, prayers at 6.50, and they enter the wards at 7 o'clock. At 10 they can have lunch of bread and cheese and coffee. Then some of them go off duty till 12.30; the others take 2.30 to 5. These latter have tea before they re-enter the wards. The Sisters and staff nurses have on alternate days from 2.30 to 5, and from 5 for the rest of the evening. One Sunday the off time is from 10 to 3; the next from 3 to 10. On the Sunday when probationers are free in the morning they are expected to attend the service in the chapel."

"Is it imperative that all the nurses should belong to the Church of England?"

"Not necessarily. I have no objection to receiving Dissenters or Roman Catholics if they are otherwise suitable."

"How long is the yearly holiday?"

"The nurses have three weeks' annual leave, of which 14 days is taken the first year at the end of the first six months. The Sisters and private nurses get four weeks. Both Sisters and nurses have a whole day once a month when feasible."

### The Private Staff.

"You allude to the private staff. Do the private nurses assist in the hospital?"

"Not unless I have a number of nurses laid up. The two institutions, although almost under one roof—the Home is only a stone's throw away, and is connected by a passage—are perfectly distinct, except that in the event of any special operation the private nurses may witness it if they wish, and when they are not at a case they may attend the nurses' lectures. They frequently do so, as it keeps their knowledge up to date. Some are doing so now. The lectures are given once a week to the junior nurses, and also to the seniors, always by some member of the staff. The Sisters instruct them in the wards in practical nursing."

"Are the probationers paid during the first year?"

"No; but they are given complete outdoor uniform, a brown cloak and brown bonnet—a veil is added after the first twelve months—as well as the material for their ward dresses of blue check galatea, and for their aprons. They have to provide the making, and buy their own caps, cuffs, and collars. The second year, when they are called junior assistant nurses, they receive £12, and the third, when they become seniors, £16. At the end of the third year, if they obtain their certificates, they are eligible to go on to the private staff if they wish it and I think they will do well."

"Do many pass on to the private side?"

"Yes; a large number, because of the increased pay. When first I came here the private nurses were only receiving £20 a year and a percentage on their earnings, but now at the end of their third year they can double their salary, for the pay on the private staff begins at £32 and rises to £36. They also get all uniform given them except collars and cuffs. The private staff number about twenty."

### The Wards and Nurses' Quarters.

"How is the hospital staff apportioned?"

"There are 82 beds, including 12 in the children's ward. When we have more than that number the other children have to be distributed about in the adult wards. The staff consists of five sisters and 22 nurses. The special theatre nurse only holds the post for about three months at a time."

"What about night duty?"

"It is taken for three months at a time, but no probationer is put on night duty till she has been six months in the hospital. When all the wards are open there are six nurses and a Sister on duty at night. And now, perhaps, you would like to see over the building."

As I passed through the wards the matron pointed out that all the walls, which had been recently done, are painted two shades of a pretty green, and told me that she hoped in time, as funds came to hand, that the same colouring would be adopted all over the hospital. Leading off from several of the larger wards are nice covered balconies, where convalescent patients can sit out and which are much appreciated by the men because they are allowed to smoke and can watch the cricket and football in the College field. Some of these balconies have been paid for by friends who have collected subscriptions for the special purpose. One nice little ward of two beds the matron indicated as that which she generally used for her nurses when they were ill. Attached to each of the large wards is the Sister's sitting-room, one already showing signs of preparations for Christmas."

As to the nurses' quarters, they are in need of additional space, which the matron hopes to get when more money is available. In the hospital the nurses' sitting-room is also their dining-room, and the hospital and the private nurses all sleep in cubicles. Some of these have doors, and others only curtains, but all are comfortably furnished, and the utmost has been done with the limited space. There is a large stove at the end of the room, and when the weather is cold this is lighted before the nurses go to bed, so that the cubicles are adequately warmed. The windows look on to a pleasant open space behind, where there is green grass and fruit trees, and a tennis court in the field adjoining, which the nurses are allowed to use. Finally, after seeing the up-to-date little operating-theatre, we visited the chapel, where there is ample room for the convalescent patients, domestic staff, and the nurses.

### Everybody's Opinion.

### TAXING THE UNIFORM.

"A Somersetshire Nurse" writes:—There is a great deal of talk about the registration of midwives, nurses, etc., but, to my mind, there is a more important matter to be dealt with—viz., the employment by the public of half-trained nurses. These women don the uniform, go to their cases, demand the same fees as the nurses who have given their services and worked hard for three years. This is distinctly wrong. Why should not all uniform worn by trained nurses, on production of their certificate, be taxed? No nurse who values her profession would object to pay. In no other profession is uniform allowed till the candidate has passed the necessary test and training—the soldier and the sailor have to earn their's, the barrister does not wear a wig and gown until he has been called to the Bar. Let those of us who value our profession and high calling see that this matter is righted.

[This suggestion, we fear, is quite impracticable. It would afford no real protection to nurses, and the tax would fall upon the wrong shoulders.—Ed. The Hospital.]

### WOMEN ON BOARDS OF HOSPITAL MANAGEMENT.

"A Matron" writes: May I say a few words, from a matron's point of view, concerning your article on this subject? If the right kind of women are appointed to form a ladies' committee I think they are most helpful, but, of course, they must have implicit confidence in their matron, and uphold her authority. Fussy, meddlesome women would be disastrous anywhere, and would create quite as much mischief as lady visitors, indeed more than if they served on a committee, because in the latter form they would meet and confer together instead of visiting the wards singly. In the hospitals in which I worked we always had a ladies' committee, and they helped greatly in interesting their friends in the work, and at Christmas they always provided presents for the staff and useful articles for the patients, thereby giving great assistance to the matron and sisters, whose purses are generally taxed to the utmost at these times. When I was appointed matron of a large convalescent home, I found the ladies' committee existed in name only. They were duly appointed every year, but they never met, and they took no part in the management of the institution. I did not rest until I got the ladies' committee in working order, and when they once began to meet as regularly as the general committee (men's) I found them of the greatest possible assistance. They never interfered but were always willing and ready to listen to my requirements and understood matters pertaining to the management of a large staff of servants, and of the female patients, far better than any men could do, however much they tried. They helped me at Christmas to provide presents for all the inmates and also to entertain them; and I was always thankful to have their advice and help, and could always rely upon their support. If they meet regularly, and consult with the matron, and meet the general committee, when they think necessary to bring improvements or other important matters before them, I am sure they will save the gentlemen a great deal of time and trouble, and that any institution will greatly benefit by their inspection. Certainly any hospital that has beds for women and children would benefit by having a ladies' committee composed of the right kind of women.

### DISTRICT NURSES' HOLIDAYS.

Miss K. E. Barlow, Superintendent of Queen's Nurses at Leeds, writes: The provision of suitable holidays for the staff of the Leeds District Nursing Association offers considerable difficulties. The nurses are already so full-handed that no one nurse can take on the work of another during her absence, and it is therefore necessary to employ special holiday nurses, who undertake the work of each of the regular staff in succession. But this plan is not altogether satisfactory for two reasons. In the first place, a nurse coming temporarily into a new district is not so efficient as one thoroughly conversant with the district and with the patients, so that with the best intentions the latter suffer to some extent. And secondly, the cost of providing holiday nurses, three of whom are required during the whole summer season from April to November, by the Leeds Association, is a heavy drain on funds already inadequate for the quantity of work which is to be done. It has therefore been suggested by a subscriber that in lieu of these holiday nurses, the homes should be entirely closed for four weeks in the summer. At this season the work is the lightest, and therefore the nurses would be the least missed. The staff would all have their holidays at the pleasantest time in the year, and would therefore obtain the maximum of refreshment and strength for the next year's work; while a minor but still important practical advantage would be that the absence from the home would enable the superintendents to get the homes cleaned, whitewashed, etc., more effectually than can be done when the home is fully occupied. On the other hand, there is, of course, the great difficulty that patients, some of them serious cases, would necessarily be neglected during the absence of the staff. The object of this letter is to elicit from any of your readers who may be interested in the subject, or possessed of experience in district nursing, their opinion on the question. More particularly, those responsible for the Leeds District Nursing Association wish to learn whether this plan of entirely closing the home has been tried in other cases, and with what results.

### Appointments.

City Hospital North, Liverpool.—Miss L. Harris has been appointed assistant matron, and Miss Florence Harper sister. Miss Harris was trained at Monsall Fever Hospital, Manchester, and Stockport General Infirmary. She has since been sister at the City Hospital North, Liverpool. Miss Harper was trained at the Mill Road Infirmary, Liverpool.

Durham Isolation Hospital.—Miss A. A. Mould has been appointed superintendent nurse. She was trained at Brownlow Hill Infirmary, Liverpool, and the Liverpool Fever Hospital, and has since done private nursing in Newcastle-on-Tyne.

Fulham Infirmary, London.—Miss Edith Amy Tucker has been appointed second assistant matron. She was trained at the Poplar and Stepney Sick Asylum, where she has since been ward sister, theatre sister, and night superintendent.

Hackney Union Infirmary.—Miss Annie Oakes Waller has been appointed charge nurse. She was trained at St. George's Union Infirmary, London, and has since been private nurse at the Southsea Nursing Institute, charge nurse at Portsmouth Parish Infirmary, staff nurse at Luton Isolation Hospital, and nurse at Lewes Union Infirmary.

Lancaster Sanatorium.—Miss A. Meeson has been appointed sister. She was trained at the Mill Road Infirmary, Liverpool, where she has since been holiday sister.

Pontypridd Workhouse Infirmary.—Mrs. Ella Scott Davies has been appointed charge nurse. She was trained at St. George's-in-the-East Infirmary, Wapping, London. She has since been staff nurse in the same institution.

Queen Charlotte's Lying-in Hospital, London.—Miss Mary Elizabeth Davies has been appointed matron. She was trained at King's College Hospital, London, and the City of London Lying-in Hospital. She has since been sister, and assistant matron and home sister at University College Hospital, London.

Royal Halifax Infirmary.—Miss Miriam Ashley has been appointed assistant matron. She was trained at the General Hospital, Birmingham, where she was afterwards sister successively of a medical ward, a surgical ward and the operating theatre. She has since been night superintendent of the Royal Infirmary, Bradford.

St. Albans Hospital, Herts.—Miss Agnes Fry has been appointed staff nurse. She was trained at Southport Infirmary.

Up-Country Nursing Association for Europeans in India.—Miss Bertha Owen has been appointed nurse. She was trained at St. John's Hospital, Lewisham, and St. John's Maternity Home, Chelsea. She has since done private and district nursing and been charge nurse at Hexham Infirmary.

### Presentations.

Addenbrooke's Hospital, Cambridge.—On Monday last, Miss Margaret Morgan, on her retirement from the matronship of Addenbrooke's Hospital, Cambridge, was presented with a sapphire and diamond ring, from the members of her past and present nursing staff, in appreciation of her work for and amongst them.

Bourton on the Water Cottage Hospital.—Miss E. J. Whiteside, matron of Bourton on the Water Cottage Hospital, has been the recipient from the committee of a solid silver inkstand, in recognition of the conscientious way in which she has performed her duties.

Bristol Children's Hospital.—Miss Neale, who is leaving the Royal Hospital for Sick Children and Women at Bristol to be married, has been presented by the matron and nursing staff with a handsome electro-plated teapot in appreciation of her worth and loyal services.

Crumpsall Infirmary, Manchester—Miss Mackay, who has resigned her post as sister, after 24 years' service at Crumpsall Infirmary, and retired from work, has been presented by the lady superintendent, on behalf of the nursing staff, with a handsome dressing-case in token of their esteem.

Epsom Infirmary. — Mrs. Richards, on leaving her appointment as charge nurse at Epsom Infirmary, to take up new duties in Wales, has been presented with a dressing-case by the master and matron, and a gold bracelet by the charge nurses and probationers of the infirmary in token of their esteem.

Selby Cottage, Hospital.—Nurse Edginton, of the Selby Cottage Hospital, has been presented with a tea and coffee service in recognition of the care and attention bestowed by her on a sailor belonging to Barton, who sustained severe injuries at Selby some time ago.

### Help the Nurses to Help Themselves.

"The Hospital" Convalescent Fund.—The object of this fund is to provide rest for weary and needy workers during convalescence after illness amidst suitable surroundings, without any anxiety about ways and means. Since its establishment many tired and delicate nurses have enjoyed a much-needed change of air such as they could not possibly have secured for themselves without help. Experience has proved that it is better to let the nurses have a choice of locality than to send them to one settled place, and nurses are accordingly sent to all parts of the country. Contributions which would increase the field of usefulness are invited by the Hon. Secretary, care of the Editor of The Hospital.

The Junius S. Morgan Benevolent Fund.—This is an auxiliary to the Royal National Pension Fund for Nurses. It was founded by nurses as a memorial to the late Junius S. Morgan, and raised to handsome proportions by the Morgan family and many other friends to nurses. An influential committee (many members of which are hospital matrons) supervises the investigation of claims, and devotes much care and attention to the relief of necessitous members of the Pension Fund. Secretary, Mrs. Bretland Farmer.

### "The Hospital" Convalescent Fund.

The hon. sec. acknowledges with grateful thanks the receipt of 2s. 6d. from Nurse E. Jones and 10s. from "Sister."

### Christmas Literature.

### BOUND MAGAZINES.

"The Girl's Own Annual" (8s.), published by the Religious Tract Society, is a gift book which none can surpass. The present volume, besides stories and papers by all the best writers, has some charming engravings of the most celebrated pictures of Greuze. "The Leisure Hour" (7s. 6d.), also published by the Religious Tract Society is as attractive as it always is, and would make a delightful Christmas gift. "Little Folks," Cassell and Co. (3s. 6d.), as it cannot fail to please, is a safe venture for any present-giver. "The Wonder Book" (3s. 6d.), published by Ward, Lock, and Co., is an annual for boys and girls. Those children who had a copy given them last year will probably count upon a new one for 1905, and they will be wise in their generation, for the new volume is full of the sort of tales the young ones love, with fascinating pictures. "C. B. Fry's Magazine" (6s.), George Newnes, Ltd., is the first volume of the new magazine of sports and outdoor life, and it should be greatly in demand as a gift for schoolboys and young men, whose tastes are sometimes difficult to cater for. The general excellence of the magazine is beyond dispute. Another capital present for boys is 'The Captain' (6s.), Vol. 11, by the same publishers. Not only are there plenty of stories of adventure and school life, but philatelics, athletics, photography, and natural history all find a place, and the illustrations are good. The new bound volume of "Sunday" (3s.), published by Wells, Gardner, Darton, and Co., is as full of good things as usual, and would make a splendid present. There are two serial stories—one by E. Everett Green—many short stories and good illustrations, all bound in an attractive cover.

### MISCELLANEOUS BOOKS.

Lovers of the Classics will appreciate "The Journal to Stella" (3s. 6d.), published by George Newnes, Limited, an attractive edition of Dean Swift's celebrated correspondence. The dainty volume on thin paper, bound in soft red leather, should form an acceptable gift. "A Bride from the Bush," by E. W. Hornung, and "The Wyvern Mystery," by Sheridan Le Fanu, are good sixpenny books published by the same firm. The charming fairy tales of Mr. George Macdonald, well-known to the children of the last generation, are now happily being republished by Mr. Arthur Fifield at 6d., or in better binding at 1s. or 2s. each. "The Light Princess" and "The Giant's Heart and the Golden Key" are the first two of the series followed by "The Shadows" and "Little Daylight." The illustrations are the original ones by Mr. Arthur Hughes drawn 40 years ago. For the convenience of those who wish to send a trifle to children by post, Messrs. Dean have brought out the new Postal Toy Book (1d.). It is in the form of a letter card, containing a coloured and illustrated booklet of nursery rhymes or stories, and space for writing a few lines. A penny stamp is affixed to the card after addressing it. Messrs Pears again present a marvellous sixpenn'orth for Christmas. There are three coloured separate pictures, "Alice in Wonderland," "A Lively Measure," and "Fruit and Flowers," the letter-press being largely devoted to extracts from the Christmas writings of Charles Dickens, with illustrations by John Leech, Charles Green, and Frank Dadd. It is quite a British production; all the books and plates have been entirely printed in England. The "Windsor Magazine" (1s.) for December, is amongst the best Christmas numbers. Fact and fiction are well intermingled, there are numerous illustrations, and amongst the writers are Rudyard Kipling, Rider Haggard, Eden Philpotts, Ethel Turner, Barry Pain, and Israel Zangwill. "Cassell's Christmas Magazine" makes a present to its readers of a copy of a picture by John Pettie, entitled "Two Strings to her Bow." Amongst the varied contents are stories by Max Pemberton, Pett Ridge, Robert Barr, Guy Boothby, and William le Queux.

### Echoes from the Outside World.

### Movements of Royalty.

The King and Queen concluded their visit to Culford Hall on Saturday. Prior to their departure for London they spent some time in Bury St. Edmunds, the streets of the town being gaily decorated and thronged with enthusiastic spectators. Near St. Mary's Church 2,000 school children sang the National Anthem. Their Majesties entering St. Mary's examined the tablet in the South Chapel to Mary Tudor, daughter of Henry VII., and afterwards Queen of France, and also the memorial window to the latter erected by the late Queen Victoria in 1881. The King suggested that the memorial stone of Mary Tudor, which is let into the floor of the sacrarium, should be made more prominent, and at the same time protected by having some Pointed Gothic carving as a coping post round it. He also asked for the story of the great monumental tablets in the church, and inspected the memorials of the Suffolk Regiment. A local Crimean hero was presented to the King and Queen in the church. . . . On Monday the King held at Buckingham Palace the final Court function of the year, and invested with the insignias of various Orders some 60 gentlemen whose respective distinctions have already been gazetted. The Duke of Connaught and the principal State officers attended the ceremony, which took place in the Throne Room. The King wore Field-Marshal's uniform, and the investiture lasted less than half an hour. On Monday the Queen selected her Christmas gifts at Buckingham Palace, when two rooms adjoining her apartments were filled with a variety of novel and costly goods.

### Their Majesties' Courts.

It is officially announced that the King and Queen will hold a series of Courts at Buckingham Palace during the coming season, at which presentations of ladies to their Majesties will be made. Two Courts will be held before Easter, probably during the month of February. A lady who makes a presentation to their Majesties must be personally acquainted with and responsible for the lady she presents. She must herself attend the Court, and cannot present more than one lady in addition to her daughter or daughter-in-law. If it should not be convenient for a lady to attend or be present at the particular Court to which she is summoned, it will be open to her to make her excuses to the Lord Chamberlain in writing, when her name can, if desired, and if possible, be transferred to another list. The Courts will be held in the evening. The dress regulations for ladies are, full Court dress with feathers and trains.

### The War in the Far East.

A striking account has been given of the fighting at 203-Mètre Hill by Commander Mizzenoff, who, after being wounded in the struggle, reached Chi-fu in a sailing-boat last week. He says that the Japanese were compelled to climb up the slopes—in many cases without themselves discharging a shot—in the face of a murderous fire from rifles and machine-guns. The attacking force went down in squads and companies, but there were always others pressing unswervingly forward. Sometimes the fighting was hand to hand. Nine standard-bearers, who tried to place the flag on the crest of the hill, were killed before the task was accomplished. The General states that there are 16,000 Russian troops living in the forts, and that they have fair intervals of repose. The rations, he adds, are sufficient for three months, and the ammunition for a much longer period. . . . A correspondence has taken place between General Stössel and General Nogi at Port Arthur, with respect to the alleged bombardment by the Japanese of Russian hospitals. In reply to the complaint of the Russian commander, General Nogi says that the Japanese have never once intentionally trained their guns on any building or ship flying the Red Cross, but that a large part of the interior of the fortress was invisible from their gun position, and it was impossible to guarantee that no shell should reach unintended places. . . . According to official reports from Tokio the Japanese caused a great explosion on Sunday on the breastwork of the north fort of Tunkeekwanshan. The Russians offered the stoutest resistance, but, after several hours desperate fighting, the fort was captured. Five field-guns, two machine-guns, and a large quantity of ammunition were captured in the fort, where about 40 Russian corpses were found. . . . Admiral Togo has sent to the Japanese capital a detailed account of the attacks by torpedo-boats on the battleship *Sevastopol.* He affirms that there is no reason left for doubt as to the Russian ships in the harbour being totally unfit for service.

### The New Master Gunner.

Lord Roberts, who has recently returned from a long tour in South Africa, has been appointed Master Gunner of St. James's Park, in succession to the late General Sir Collingwood Dickson. Master Gunner of St. James's Park is a purely honorary title given to distinguished soldiers, frequently those holding the Victoria Cross. In olden times the Master Gunner had important functions to perform, as it was his duty to be responsible for the royal salutes in the Park. For this duty four shillings and three pence a day was given to him from an old Court Fund, and the sum is still paid to the holder of the office.

### Funeral of Mr. Kruger.

The mortal remains of Paul Kruger, the last President of the Transvaal Republic, were laid to rest on Friday, the day being chosen because it was Dingaan's Day, the great Boer national festival. An impressive religious service was held in the forenoon in the open air in the grounds attached to the Dopper Church, opposite the late Presidency. About 2,000 burghers were present, and three Dutch ministers addressed the congregation. Mr. Schalk-Burger, General De Wet, and General Botha also spoke. General Botha contended that Mr. Kruger had filled in the Afrikander nation the place which Moses had filled among the Israelites. His ideal had been to create a great nation under the Sand River Convention from the Vaal River northwards, from sea to sea. He urged the South African people to carry out this idea, while at the same time maintaining their loyalty to the new Government.

### New Play at the Prince of Wales's Theatre.

On Saturday evening a new musical comedy, called "Lady Madcap," by Mr. Paul Rubens and Colonel Newnham-Davis, was produced at the Prince of Wales's Theatre, with every promise of sustained success. The favourable reception was due to the excellence of the piece and the admirable acting and singing of the players. The scene of the comedy is Egbert Castle, East Anglia, to which the regiment of East Anglian Hussars are invited at the instance of Lady Betty Claridge, otherwise "Lady Madcap." She is in love with a very comic hussar, Trooper Smith, who is really a millionaire sailing under false colours. There is also a sham millionaire who is really a scheming adventurer who aspires to marry the heroine. These are the three principal characters, but the others contribute more or less to the fun, which is very diverting and unobjectionable. Several of the songs are sure to be heard during the holiday season. The Queen was present at the second performance on Monday.

### A Book and its Story.

A DESERT ROMANCE.*

So much has been written in praise of Mr. Hichens' new book that it is perhaps unnecessary to express any further appreciation of it here except to say that "The Garden of Allah" is a distinct advance on anything that he has previously produced. It is a unique and daringly-conceived novel, remarkable for its powerful characterisation and exquisite pen pictures of the Sahara Desert, pictures so penetrated with the spirit of place that they exercise a spell over the reader almost as weirdly fascinating as that of the actual region itself.

Domini Rens, the heroine, is an extraordinarily strong delineation. She is thirty-two when her story begins. "Domini was unmarried, and in a singularly independent—some might have thought a singularly lonely—situation. Her father, Lord Rens, had recently died, leaving Domini, who was his only child, a large fortune. His life had been a curious and a tragic one. Lady Rens, Domini's mother, had been a great beauty of a gipsy type, the daughter of a Hungarian mother and of Sir Henry Arlworth, one of the most prominent and ardent English Catholics of his day . . . Lady Rens, who was not clever, although she was universally considered to have the face of a muse, shared the family ardour for the church, but was far too fond of the world to leave it. While she was very young she met Lord Rens, a Lifeguardsman of twenty-six, who called himself a Protestant, but who was really quite happy without any faith. He fell madly in love with her, and in order to marry her became a Catholic, and even a very devout one, aiding his wife's church by every means in his power, giving large sums to Catholic charities, and working with almost fiery zeal for the spread of Catholicism in England." The change of communion was adopted by Lord Rens solely out of devotion to his wife who, "when Domini was nineteen had suddenly thrown all her principles to the winds, and runaway with a Hungarian musician who had made a furore one season in London by his marvellous violin-playing." Therefore it is not surprising to read that "her husband, stricken in soul, and also wounded almost to the death in his pride, abandoned abruptly the religion of the woman who had converted and betrayed him." Domini's home life from this time was one of intense unhappiness. Her father, relieved from all religious restraints, became an embittered and cynical man. He failed to pervert Domini to his Atheistic attitude towards life which his own miseries had led him to adopt. When Domini was twenty-one she lost a valued friend and director in Father Arlworth, "who had a subtle understanding of human nature." His loss was a great one to her, for he understood her temperament, and he saw that the effect of her mother's conduct was to make her slightly hard, and he knew that her nature required peace in its environment to bring it to a normal condition after the shock she had sustained. Instead of this we read: "She lived with her father, and drifted into the unthinking worldliness of her order. Her home was far from ideal. Yet she would not marry. The wreck of her parents' domestic life had rendered her distrustful of human relations. She had seen something of the terror of love, and could not, like other women, regard it as safety and as sweetness. So she put it from her, and strove to fill her life with all those better things which men and women grasp, as the Chinese grasp the opium pipe, those things which lull our comprehension of realities to sleep." This was the condition of things when Domini's father died. Left alone, with a future to face, and a past filled with memories upon which she could not dwell, she felt the imperative need of change—some change which would take her far from her present surroundings into a wider and more spacious environment. While still in perplexity as to her ultimate destination, she starts with her maid for the south of Europe, and while there takes the sudden determination to cross over to Africa and go to Beni-Mora. "Idly she fancied that perhaps in the sunny solitude of Beni-Mora, far from all the friends and reminiscences of her old life, she might learn to understand herself. How? She did not know. She did not seek to know. Here was a vain pilgrimage, as many pilgrimages are in this world—the journey of a searcher who knew not what she sought." During the long railway journey which intervened before she reached her destination she had time to take in the impressions which the strange, desolate country through which they were passing made upon her mind."

"She began with a faint excitement to realise . . . that somewhere beyond the broken and near horizon line towards which the train was creeping lay the great desert, her destination, with its foul sands and desolate cities, its sunburnt tribes of workers, its robbers, warriors, and priests, its ethereal mysteries of mirage, its tragic splendours of colours, of tempest and heat." Then comes a powerful description of the gradual approach to the desert itself; "a sense of leaving all things behind had stolen over her. She was really fatigued by travel and by want of sleep, but she did not know it. Lying back in her seat she watched the great change that was coming over the land. It seemed as if God were putting forth His hand to withdraw gradually all things of His creation, all the furniture He had put into the great palace of the world; as if He meant to leave it utterly empty and naked. First He took the rich and shaggy grass and all the little flowers that bloomed modestly in it. Then He drew away the orange groves, the oleander and the apricot trees, the faithful eucalyptus, with its pale stems and tressy foliage, the sweet waters that fertilised the soil . . . the tufted plants and giant reeds that crowd where water is." As the outlook became still wilder and more barren she felt that "she had passed the boundary of the world God had created, and come into some other place upon which He had never looked, and of which He had no knowledge." And yet, as they neared Beni-Mora Domini realised for the first time in her life the power of colour as an appeal to the imagination. It was towards sunset that they neared the city in the desert. "They were near Beni-Mora now. Its palms appeared far off, and in the midst of them a snow-white tower. The Sahara lay around and beyond it. A long spur of rose-coloured mountains stretched away towards the north. . . . In this pageant of the East she saw the naked soul of Africa—no faded, gentle thing, fearful of being seen, fearful of being known and understood, but a phenomenon vital, bold, gorgeous, like the sound of a trumpet pealing a great *réveillé*. The fatigue in her died. . . . In the huge spaces of the Sahara her soul seemed to hear the footsteps of Freedom treading towards the south, and all her bitterness of ennui, all her questionings and doubts were swept away on the keen desert wind into the endless plains." To what destiny Domini was hastening it is not our province to divulge. But for pathos, mystery, and passion its development is unique in the annals of past or present day novels. We have given prominence to her as a heroine of so an unusual type that she dominates the story from first to last. The few other characters in it are equally conspicuous for originality of treatment, and we regret that space does not permit of any notice of them. They must be studied in "The Garden of Allah" to be fully appreciated.

* "The Garden of Allah." By Robert Hichens. Methuen. 6s.

### Notes and Queries.

**FOR REGULATIONS SEE PAGE 12.**

*L.O.S. Certificate.*

(86) I am a trained nurse and wish to take the L.O.S. certificate. Will you please advise me as to where, when, and by whom the examinations are held? What is the minimum number of cases which must be attended, and would preparation by a medical man serve in lieu of training at a maternity hospital?—*M. H. L.*

Write for full particulars to the Secretary, the London Obstetrical Society, 20 Hanover Square, London, W.

Will you kindly tell me when the examinations for the L.O.S. are held, and also give me the names of two or three places where training for it can be obtained at a low fee?—*Matron.*

See reply to *M. H. L.*, and also "The Nursing Profession: How and Where to Train," for a full list of training schools.

*Private Nursing Home.*

(87) I shall be much obliged if you will kindly give me the address of a private nursing home in London or the suburbs.—*Sister Catherine.*

We cannot give the names and addresses of private institutions

*Massage.*

(88) Will you kindly tell me where I could have lessons in massage, either in London, Bedford, Cambridge or Leicester? How long would the course be, and what would be the cost?—*A. B.*

The Society of Trained Masseuses, 12 Buckingham Street, Strand, can probably help you.

*Naval Nurse.*

(89) Will you kindly give me the name of the authorities to whom I should apply for nursing appointment in the Navy?—*Rita.*

For information respecting Queen Alexandra's Royal Naval Nursing Service, write the Director-General, Medical Department of the Navy, 18 Victoria Street, London, S.W.

*Addresses.*

(90) Will you please give me the address of the Colonial and Foreign Nurses' Association, and of the British Nurses' Association?—*Nurse P.*

The address of the Colonial Nursing Association is Imperial Institute, London, S.W.; and of the Royal British Nurses' Association, 10 Orchard Street, London, W.

*Dispensing.*

(91) I have passed the first Arts Examination in the Royal University of Ireland and would like to know if this will qualify me for undertaking the post of dispenser to a medical man.—*C. W.*

Write to the Secretary, the Pharmaceutical Society, 17 Bloomsbury Square, London, W.C., for list of examining bodies whose certificates are accepted as qualifying their owner to dispense.

*Astigmatism.*

(92) Will you kindly tell me if an astigmatism, which compels the constant use of spectacles, will prevent my being trained as a nurse?—*F. H. E.*

Not necessarily, but the medical man who examines you for admission as probationer will tell you.

*Registration.*

(93) Will you kindly tell me how a nurse holding the certificate of the L.O.S. may register herself as midwife?—*L. W.*

Write to the Central Midwives Board, 6 Suffolk Street, Pall Mall.

*Asylums, South Africa.*

(94) Will you kindly give me a list of the asylums round Capetown and Durban?—*Nurse R.*

Write to the Medical Superintendent, Valkenberg Hospital for the Insane, Mowbray, Capetown, for particulars relating to asylums in South Africa.

*Benefit Fund.*

(95) I am anxious to subscribe to a fund which benefits old or sick nurses?—*Peg.*

Write to the secretary of the Royal National Pension Fund for Nurses, 28 Finsbury Pavement, London, E.C.

**Handbooks for Nurses.**

| | Post Free. |
|---|---|
| "The Nursing Profession: How and Where to Train" ... | 2s. 4d. |
| "The Nurses' Pronouncing Dictionary" ... ... ... | 2s. 0d. |
| "Nursing: its Theory and Practice" (Lewis) ... ... | 3s. 6d. |
| "Surgical Bandaging and Dressings" ... ... ... | 2s. 0d. |
| "A Complete Handbook of Midwifery" (Watson) ... | 6s. 4d. |

Of all booksellers or of The Scientific Press, Limited, 28 & 29 Southampton Street, Strand, London, W.C.

### For Reading to the Sick.

CHRISTMAS.

O Christ, Redeemer of our race,
Thou Brightness of the Father's face,
Of Him, and with Him ever one,
Ere times and seasons had begun.

Thou art that very Light of Light,
Unfailing Hope in Sin's dark night,
Hear Thou the prayers Thy people pray
The wide world o'er, this blessed day.

To-day, as year by year, its light
Sheds o'er the world a radiance bright,
The precious truth is echoed on,
"'Tis Thou hast saved us, Thou alone."

Thou from the Father's throne didst come
To call his vanished children home;
And Heaven, and earth, and sea, and shore,
His love who sent Thee here adore.

*From the Latin.*

We read in the Epistle to the Hebrews that Christ, "though He were a Son, yet learned He obedience by the things which He suffered; and being made perfect He became the Author of eternal salvation unto all them that obey Him." We are here plainly told by inspiration that one of the long lessons of our Saviour's life was obedience, learnt in the school of suffering; and in this, as in all other things, He left us an example that we should follow His steps.

In heaven obedience was unknown to the Son of God, for His will and His Father's will were one; but on earth, where He lived a human life as the Son of Man for our sakes, and was subject to all the contradiction of sinners against Himself, He had to learn the obedience which God exacts from human imperfection. "I came," He said, "not to do Mine own will, but the will of Him that sent Me."

Pain and suffering, whether physical or mental, do not necessarily lift our eyes above this earth, nor open to us the visions of the unseen world; but pain and suffering borne for Christ's sake, and in His company, transform all of Time into the light of Eternity, and renew the inward man day by day.

We often see but little outward fruit in the servants of Christ; the bed of pain or the humble path of an obscure life may leave small token to the world of the saintly spirit which overcame; but the light which guided that lowly pilgrim was the true light, and its rays have kindled many a spark which shall never be quenched.

*Mrs. C. G. Campbell.*

PRAYER.

O! Eternal Light, shine into our hearts. O! Eternal Power be Thou our support. Eternal pity, have mercy upon us. Grant unto us that with all our hearts, and minds, and strength, we may evermore seek Thy face; and finally bring us in Thy infinite mercy to Thy holy Presence. So strengthen our weakness that following the steps of Thy Blessed Son we may obtain Thy mercy and enter into Thy promised joy. Amen.

*Alcuin*, A.D. 780.

